# Uncovering Transcriptional Responses to Fractional Gravity in *Arabidopsis* Roots

**DOI:** 10.3390/life11101010

**Published:** 2021-09-24

**Authors:** James Sheppard, Eric S. Land, Tiffany Aurora Toennisson, Colleen J. Doherty, Imara Y. Perera

**Affiliations:** 1 Department of Molecular and Structural Biochemistry, North Carolina State University, Raleigh, NC 27607, USA; jvsheppa@ncsu.edu (J.S.); cjdohert@ncsu.edu (C.J.D.); 2 Department of Plant and Microbial Biology, North Carolina State University, Raleigh, NC 27607, USA; esland@ncsu.edu (E.S.L.); tatoenni@ncsu.edu (T.A.T.)

**Keywords:** spaceflight, fractional gravity, RNA-seq, Arabidopsis, gene expression, heat shock proteins

## Abstract

Although many reports characterize the transcriptional response of *Arabidopsis* seedlings to microgravity, few investigate the effect of partial or fractional gravity on gene expression. Understanding plant responses to fractional gravity is relevant for plant growth on lunar and Martian surfaces. The plant signaling flight experiment utilized the European Modular Cultivation System (EMCS) onboard the International Space Station (ISS). The EMCS consisted of two rotors within a controlled chamber allowing for two experimental conditions, microgravity (stationary rotor) and simulated gravity in space. Seedlings were grown for 5 days under continuous light in seed cassettes. The arrangement of the seed cassettes within each experimental container results in a gradient of fractional *g* (in the spinning rotor). To investigate whether gene expression patterns are sensitive to fractional *g*, we carried out transcriptional profiling of root samples exposed to microgravity or partial *g* (ranging from 0.53 to 0.88 *g*). Data were analyzed using DESeq2 with fractional *g* as a continuous variable in the design model in order to query gene expression across the gravity continuum. We identified a subset of genes whose expression correlates with changes in fractional *g*. Interestingly, the most responsive genes include those encoding transcription factors, defense, and cell wall-related proteins and heat shock proteins.

## 1. Introduction

Plant cultivation will be essential during long duration space missions to supplement nutritional needs of the crew, purify air and water, and provide psychological benefits to the crew [1]. One unfamiliar stress that spaceflight and extraterrestrial environments pose to plant growth is the lack of or reduced gravity. Since both the Moon and Mars have gravitational fields that are a fraction of Earth’s gravity, partial gravity research is especially relevant to near term space exploration [2].

On Earth, slowly rotating platforms, called clinostats, can be used to simulate microgravity. However, a certain amount of mechanical stimulation occurs during clinorotation that can be difficult to disentangle from gravitational response [3]. Similarly, partial gravity can be simulated on Earth through the use of clinostats affixed with centrifuges [4], with the same limitations as simulating microgravity. True reduction of gravitational stimuli only occurs during spaceflight where the orbital acceleration of the spacecraft counteracts the pull of Earth’s gravity and renders organisms onboard the craft “weightless”. In addition to providing a ‘1 *g*’ control for flight experiments, programmable centrifuges, such as the (recently decommissioned) European Modular Cultivation System (EMCS) on the International Space Station (ISS), allowed for the generation of partial gravitational forces during spaceflight.

Plant transcriptional responses to microgravity conditions onboard spacecraft have been characterized in numerous studies [5,6,7,8,9,10,11,12,13,14]. Differing methodology and hardware choices confound direct comparisons between these studies, but *Arabidopsis* gene expression alterations in response to spaceflight have been observed for a variety of protein functional groups, including heat shock proteins (HSP)s [7,10,11], plant defense proteins [8], light response proteins [12], and cell wall proteins [9,10]. However, only a few spaceflight experiments to date have examined gene expression under fractional gravity in a systematic fashion [15,16].

The studies that have examined the effects of partial gravity during spaceflight have revealed a suite of transcriptional and physiological changes that occur with varying intensity as gravity increases from microgravity towards 1 *g*. Some physiological responses vary in a step-like fashion with increasing gravity. For example, blue light phototropism in roots is highly apparent in microgravity but decreases rapidly as soon as plants receive gravitational stimuli [17]. This physiological change is mirrored on a transcriptional level, with genes related to photosynthesis and light response decreasing as gravity increases [15]. Other genes appear to exhibit curved or J-shaped dose-response to gravitational stimuli. Herranz et al. [15] observed more differentially expressed genes (DEGs) at low (<0.1 *g*) gravity than either microgravity or higher g levels. These DEGs include many genes associated with plant stress response. Clinostat studies have also demonstrated this J-shaped response to gravitational stimuli in regard to nucleolus size—a proxy for ribosome biogenesis [4]. In this case, the nucleolar area decreased as gravity increased from simulated microgravity to 0.18 *g* then slowly increased again with increasing gravitational force.

Increasing *g*-gradients to above 1 *g*, known as hypergravity, can provide further insight on plant response to gravitational stimuli. Hypergravity induces distinct changes in plant cell wall composition, such as increasing the amount of cell wall polysaccharides and overall cell wall rigidity [18]. Hypergravity also shifts the balance between meristematic cell growth and cell proliferation, with greater cell growth but lower cell proliferation under higher *g* levels [19]. Both cell wall remodeling and cell growth alterations occur in an inverse manner to microgravity-grown plants, supporting the idea that gradients in gravitational force can alter plant structure and function in predictable patterns [18,19].

In our experiments, we investigated plant transcriptional responses along a gradient from 0.55 *g* to 1 *g* using the EMCS hardware. The independently controlled rotors on the EMCS allowed us to simulate this gradient of gravity along one rotor and compare with simultaneously grown microgravity samples on a second rotor within the same environmental chamber. We identified 101 genes which show clear patterns of response to fractional g levels. These include several transcription factors, genes involved in cell wall modification, plant defense responses and heat shock proteins. To further investigate the effects of altered gravity on key DEGs, we examined the response of select heat shock protein genes to hypergravity.

## 2. Materials and Methods

### 2.1. Flight Preparation

The plant signaling experiment was carried out in the EMCS using the TROPI-like seed cassettes previously described [20]. Whatman grade 17 CHR chromatography paper and black gridded polyethersulfone (PES) membrane (PALL Life Sciences, 65561) were cut to fit the seed cassettes. The blotter paper was pre-soaked in 0.5X Murashige and Skoog (MS) media (without sucrose), and blotter paper and membranes were sterilized by autoclaving. Seeds were surface sterilized and allowed to dry. Healthy seeds with well-developed endosperm were selected under a dissecting microscope and mounted to the gridded membrane using guar gum. Seeds were positioned with the micropyle pointing down (away from lights) with 27 seeds/membrane. Gridded membrane/blotter paper sandwiches were fixed to the base plate, the covers were attached and sealed with aluminum foil tape (3M, 425). Seed cassettes were assembled for flight at the NASA Ames research facility and loaded into experimental containers (ECs) 35 days prior to scheduled flight. Samples were transported to the ISS aboard STS-135 (launched in July 2011) and returned on SpaceX Dragon in March 2013.

### 2.2. Spaceflight

Onboard the ISS, ECs were loaded into the EMCS by the attending astronaut. The experiment was initiated by remotely controlled hydration. The plant signaling experiment was grown for 5 days under continuous light illuminated by white LEDs at the shoot side of the cassettes. Images were obtained every 6 h from cameras located within the EMCS chambers. Temperature was maintained at 24 °C with ~20% O_2_ and ethylene scrubbing. The EMCS chambers were purged before each run and CO_2_ levels gradually increased to equilibrate with the cabin. At the end of the experiment, seed cassettes were removed from the ECs and frozen immediately in the onboard −80 freezer (MELFI). The simulated gravity rotor was removed and processed first. Seed cassettes remained frozen onboard the ISS. Samples were maintained at freezing temperatures during return via Space-X Dragon, after splashdown and until delivery to the laboratory. Samples were then stored at −80 °C until processing for RNA.

### 2.3. RNA Isolation

Seed cassettes were processed one at a time. A single seed cassette was retrieved from the freezer and the cover was removed. The cassette base was placed on a cold platform and RNA-later (Ambion, Waltham, MA, USA) was added. The seedlings were separated into root and shoot tissues and stored at 4 °C in RNA-later for 24 h followed by storage at −20 °C until RNA preparation. RNA was isolated from each root sample using the RNAqueous Micro kit (Ambion, Waltham, MA, USA). RNA recovery and integrity was monitored by Bioanalyzer. Typical RNA integrity number (RIN) exceeded 9.5.

### 2.4. Illumina Sequencing

**Figure 1:**
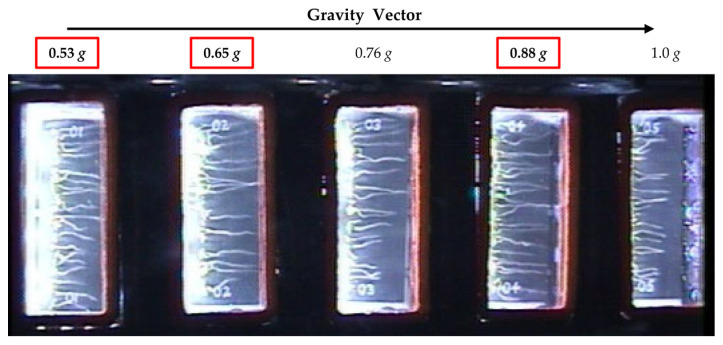
Overview image taken on day 5 during the plant signaling flight experiment showing an experimental container and the arrangement of the five seed cassettes with respect to the center of the centrifuge. Fractional *g* levels at each cassette position are indicated. The *g* levels investigated in this study are boxed in red.

Library preparation and sequencing was carried out by the Genomic Sciences Laboratory at North Carolina State University. Briefly, PolyA RNA was captured using the NEBNext Poly (A) mRNA magnetic isolation module and libraries were prepared using the ultra-directional library kit (New England Biolabs, Ipswich, MA, USA). Sequencing was carried out on 3 lanes of Illumina HiSeq2500 (125 bp single end reads). For each *g* level, we examined 4 replicate samples and 16 replicates for microgravity. For this study, we compared the fractional *g* levels, 0.53 *g*, 0.65 *g*, and 0.88 *g* (Figure 1, seed cassette positions 1, 2, and 4) and microgravity. Due to the fact that some of the seed cassettes in position 5 (1 *g*) did not maintain moisture and dried out, these cassettes were not analyzed. The 0.76 *g* level cassettes were used for a separate analysis.

### 2.5. Sequencing Analysis and Identification of Differentially Expressed Genes

Fastq files were aligned to the *Arabidopsis* TAIR10 genome using the Araport11 annotation with HiSat2 [21]. The coverage mapping rate across all samples was 97%. Counts per gene were generated using HTSeq-count [22] and the Araport11 annotation [23]. The count table was imported into R version 3.6.3 [24]. Batch corrections were performed using the svseq package, creating two surrogate variables [25]. Differentially expressed genes were determined using DESeq2 [26]. To remove genes with low expression, those with rowMeans < 1 were removed prior to differential expression analysis. We analyzed genes for a response due to fractional gravity levels using DESeq. The model statement for this analysis, Dataset (~Dataset + Gravity), treated Gravity as a continuous variable. Genes were selected as responsive to fractional *g* if they had an adjusted *p*-value of <0.001 resulting in 382 genes. All other arguments for results were left as default. Manual curation of these 382 genes yielded 101 genes that exhibited an increasing or decreasing sequential fractional *g* response between µ*g* and 0.88 *g*. These 101 genes were categorized based on their patterns of expression (Figure 1): (1) increasing or decreasing in a dose-dependent response, (2) increasing (decreasing) to a peak value, and (3) increasing or decreasing to a plateau or threshold.

### 2.6. Hypergravity Stimulation

Surface sterilized *Arabidopsis* (Col-0) seeds were stratified at 4 °C in darkness for 2 days. Then, 0.5X MS media (M576, Phytotech Labs, Lenexa, KS, USA) and solidified by 1% (*w*/*v*) Agar (Agar-M, Sigma Aldrich, St. Louis, MI, USA) was prepared in 6 well plates. Seeds were plated on media in two rows (8 seeds/row/plate) and incubated vertically in a growth chamber under long day (16 h light/8 h dark) conditions. Four-day old seedlings were subject to a hypergravity stimulus (6.5× *g*) for 4 h in the dark in a table-top centrifuge (Hermle Z300) fitted with a swinging-bucket plate rotor. Control seedlings were kept vertical in the dark for the same duration of time.

### 2.7. RNA Preparation, cDNA Synthesis, and Quantitative RT-PCR

RNA was isolated from control or hypergravity stimulated seedlings as described above and treated with rDNase I (DNAfree, Invitrogen, Waltham, MA, USA). First-strand cDNA was synthesized with random hexanucleotide primers and Multiscribe reverse transcriptase according to manufacturer’s specifications (Applied Biosystems, San Francisco, CA, USA). Quantitative reverse transcription PCR (qRT-PCR) reactions were prepared with a master-mix (Power SYBR Green, Applied Biosystems, San Francisco, CA, USA) and carried out in triplicate using cDNA equivalent to 20 ng of RNA input. Transcript abundances of the genes of interest were quantified using gene specific primers and PP2A as an endogenous control (Sequences of primers used in this study are shown in Table S3). All qRT-PCR reactions were carried out in a StepOnePlus thermal cycler (Applied Biosystems, San Francisco, CA, USA) and relative expression values were calculated according to the ΔΔCT method [27] by instrument software (StepOne v2.2.2).

## 3. Results and Discussion

### 3.1. A Subset of DEGs Show a Fractional g Response

The primary goal of the plant signaling spaceflight experiment was to identify differentially expressed genes between microgravity and the on board ‘1 *g*’ control. For this purpose, one rotor within the EMCS was kept stationary (microgravity) and the second rotor was set to a rotational speed so as to simulate ‘1 *g*’. The positioning of the seed cassettes within the EMCS rotors however, resulted in a stepwise gradient from 0.53 *g* to 1 *g* across the five seed cassettes within each experimental container in the ‘1 *g*’ control rotor as illustrated in Figure 1.

**Figure 2:**
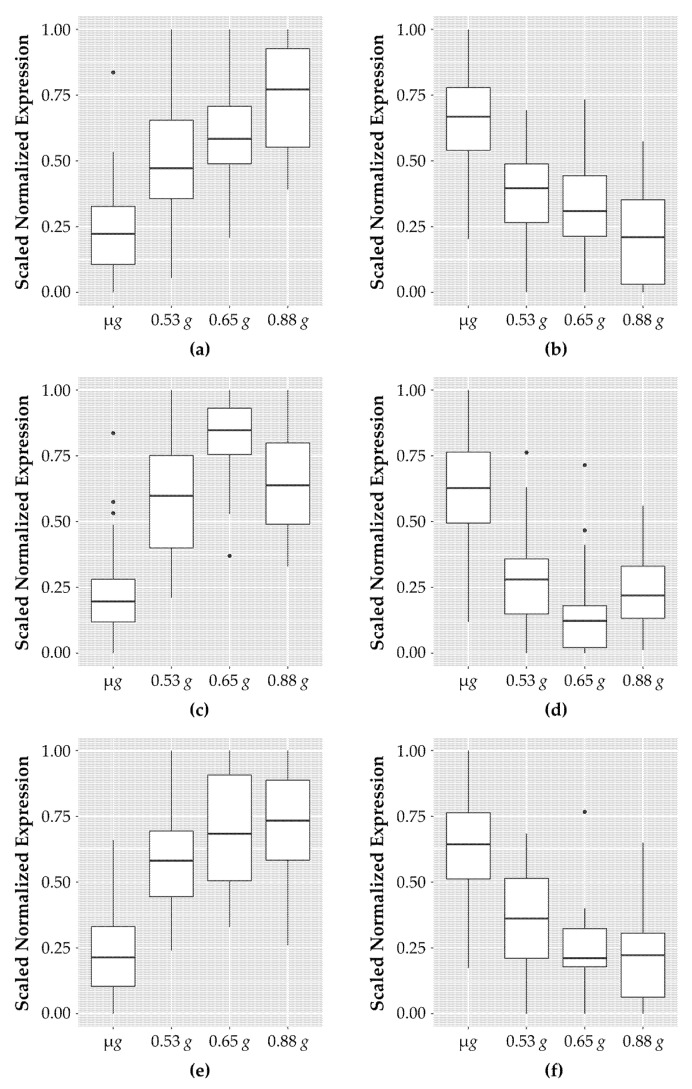
Patterns of genes which show response to fractional *g* levels. Genes for each category were scaled between 0 and 1 using the following formula: counts_normalized = (counts − min(counts))/(max(counts) − min(counts)). The boxplots indicate the expression range of the top genes in each of the samples; sixteen samples for microgravity and four samples each for the increasing fractional *g* samples (0.53 *g*, 0.65 *g*, and 0.88 *g*). (**a**) The top ten genes showing a dose-dependent increase in expression in response to increasing *g* levels; (**b**) top ten genes with a dose-dependent decrease in expression levels; (**c**) top ten genes with an increased expression of similar levels at any *g* level tested; (**d**) top ten genes with decreased expression to similar levels and any *g* level; (**e**) the nine genes that have increased expression at low *g* levels that peaks which is then reduced at the highest *g* levels tested; (**f**) the eight genes that have decreased expression at low *g* levels that increases at the highest *g* levels.

When the fractional *g* gradient across the samples in the actively spinning rotor is considered, 101 genes exhibited dose-associated expression levels that occurred in one of three different patterns of expression: (1) a dose-dependent linear increase or decrease, (2) a “peak up” or “peak down” response where expression levels peak or dip at an intermediate *g* level, and (3) a threshold response where expression levels are induced (or repressed) to a threshold *g* level. These fractional *g* responsive expression patterns derived from representative genes in each group are illustrated in Figure 2. The 101 genes along with the normalized count data for all the replicate samples are listed in Table S1.

### 3.2. Gene Ontology (GO) Classification of Fractional g DEGs

In order to identify biological functions or pathways that may be over-represented in the 101 fractional *g* responsive DEGs, the gene list was queried using AgriGO2 and ExPath2. Table 1 shows the significantly enriched (FDR, 0.05) gene categories. These genes include transcription factors, genes with chaperone function, cell wall associated genes, and genes involved in defense responses. The individual genes in each of these categories are shown in Table 2.

### 3.3. Regulation of Transcription

**Figure 3:**
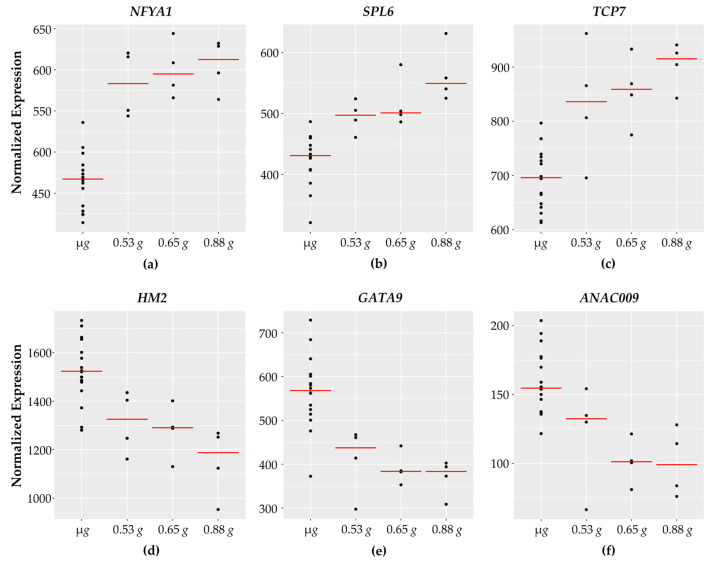
Transcription factors with fractional *g* responses. Each dot on the graph shows the normalized gene expression level for the transcription factor in the sixteen microgravity replicates (µ*g*), and the four replicates at 0.53 *g*, 0.65 *g*, and 0.88 *g*. The red line indicates the mean expression level in that gravity level. Shown are three transcription factors with a gravity-level responsive increase in transcription (**a**) *NFYA1*, (**b**) *SPL6*, and (**c**) *TCP7*. (**d**) *HM2* shows a dose-dependent decrease in transcript levels in response to increasing gravity levels. (**e**) *GATA9* and (**f**) *ANAC009* both show a threshold response, where the transcription level is reduced in response to 0.53 *g*, and reaches a plateau at 0.65 *g* and 0.88 *g*.

These genes comprise the largest functional category identified in the dataset with 20 transcription factors and coactivators. The expression of 13 transcription factors increases with increasing *g* while 7 transcriptional factors have higher expression in micro *g* and decrease with higher *g* levels. Expression patterns of representative transcription factor genes are shown in Figure 3.

The function of the majority of these transcription factors appears to be in growth and differentiation; at least 8 of the transcription factors are implicated in root development, not surprisingly, as the tissue assayed was 5-day old roots. Four of the transcription factors with a linear, dose-dependent increase in expression with increasing gravity levels, WOX4, MYB88, SPL6, and TCP7 [28,29,30,31], are involved in developmental progression, maintenance, patterning, and growth. SPL6 may also play a role in plant responses to bacterial pathogens and was shown to activate a subset of defense related genes [32]. Additionally, MYB88 has been associated with abiotic stress responses [33]. NAC and bHLH transcription factors are generally associated with environmental stress responses. However, the specific functions of these particular transcription factors, NAC97 and bHLH99, have not been experimentally validated in *Arabidopsis*. CDF2 is implicated in blue light signaling and was shown to regulate miRNA accumulation through direct transcriptional control as well as at a post-transcriptional level [34]. 

The transcription factor LNK1 shows a peak induction at 0.65 *g* and then reduces expression at 0.88 *g*. LNK1 is one of four night light inducible and clock-regulated (LNKs) proteins in *Arabidopsis* involved in integrating light and circadian signaling [35]. 

Six transcription factors are upregulated in response to gravity in a threshold-type manner. These genes are induced at 0.53 *g*, compared to the microgravity samples. However, their expression seems to plateau between the next two *g* levels (0.65 *g* and 0.88 *g*). Four of these transcription factors, AGL12, MYB53, NFYA1, and OBP3, are associated with growth and development and are expressed highly in root tissue. MYB53 is a member of a small subgroup of R2R3 MYB transcription factors. MYB53 expression was induced within 6 h after gravistimulation in the region of the root where lateral roots initiate [36]. NFYA1 plays a role in modulating growth in response to salt stress [37]. OBP3s induced in roots in response to auxin and salicylic acid (SA) and overexpression of OBP3 results in altered root and root hair development [38]. A specific role for bHLH27 has not been functionally verified. The heat stress transcription factor HSFA3 is induced in response to heat stress and regulates a suite of targets that improve tolerance to heat and oxidative stress [39,40].

Six of the transcription factors showed expression patterns decreasing with increasing *g*. Of the three genes with a linear, dose-dependent decrease in expression, HM2 is involved in development and patterning and maintaining organization of the meristem [44]. Although homeodomain transcriptional factor family proteins are also frequently involved with development and patterning, little information is known or experimentally validated for the specific function of ZFHD3. EIL2 is associated with ethylene signaling [45]. 

Three transcription factors decrease and reach a steady level with increasing *g*. ANAC009, (also known as FEZ), is a regulator of cell division and development in the root cap [46]. GRF3 is a regulator of developmental responses, tissue development, and differentiation [47]. GRF3 also serves as an integrator connecting biotic and abiotic stress responses with developmental progression [48]. GATA9 is a class IV Zn-finger transcription factor. Although the 28 member GATA transcription factor family in *Arabidopsis* is broadly associated with light and nutrient responses, functional validation of a specific role for GATA9 has not been described [49]. 

### 3.4. Transcription Factor Binding Sites and Putative Targets

We investigated whether target binding sites had been identified for any of the transcription factors reported here. Surprisingly, we found only five of the TFs have directly assayed motifs [50,51,52]. We next checked for overlap between putative targets and genes in the 101 list. The target binding sequence of OBP3 has low information content and is present in the promoter region of over 15,000 genes [52]. Of these 24 putative targets are present in the 101 gene list (Tables S2 and S3). These genes include other transcription factors (MYB88, GRF3, GATA9, CDF2, HM2 and TCP7) and several cell wall related genes. Six genes are TCP7 targets, including two transcription factors HSFA3 and NFYA1. GATA9 also targets NFY1A and PGL3 is a target of MYB88 (Tables S2 and S3).

It is noteworthy, that several of the transcription factors with a response to fractional g have not been functionally studied in Arabidopsis despite the fact that they are members of well-characterized transcription factor families (e.g., GATA, MYB, bHLH, NAC). Perhaps their response to fractional g can provide insights into their functions and regulatory targets.

### 3.5. Defense Responses 

**Figure 4:**
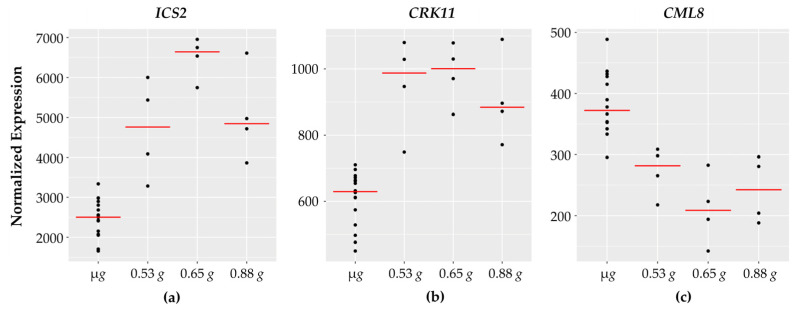
Defense related genes with fractional *g* responses. Each dot on the graph shows the normalized gene expression level for the transcription factor in the sixteen microgravity replicates (µ*g*), and the four replicates at 0.53 *g*, 0.65 *g*, and 0.88 *g*. The red line indicates the mean expression level in that gravity level. Shown are three representative genes involved in defense: (**a**) *ICS2* and (**b**) *CRK11* expression increases with peak expression at 0.65 *g* while (**c**) *CML8* expression levels decrease with a dip at 0.65 *g*.

Ten out of the twelve genes implicated in biotic interactions and defense responses show increased expression across the fractional *g* gradient while two of them decrease with increasing *g*. Examples of the defense related genes are shown in Figure 4.

Among the defense related genes whose expression increases with *g* levels are two Ca^2+^ channels, CNGC19 and OSCA1.3. Both of these are implicated in PAMP-triggered immunity. CNGC19 was rapidly induced in *Arabidopsis* roots in response to fungal elicitors and immune responses were compromised in *cngc19* mutants [53]. OSCA1.3 was shown to be rapidly phosphorylated in response to PAMP treatment and is implicated in early immune signaling [54]. Also in this group are two receptor like kinases, the cysteine rich receptor like kinase, CRK11, and a leucine rich receptor kinase (LRR kinase). Expression of *CRK11* was shown to increase rapidly in response to Ozone (O_3_) as well as various bacterial and fungal elicitors [55]. SGT1A is a co-chaperone protein that is induced upon pathogen infection [56] and is involved in stabilizing JA receptor complexes [57]. Additionally, isochorismate synthase (ICS1) catalyzes the first step in SA biosynthesis [58].

Interestingly, expression of *CML8* (which encodes for an atypical calmodulin like protein) decreases with increasing *g*. CML8 was reported to be a positive regulator of SA-mediated plant immunity to bacterial pathogens. *CML8* gene expression was rapidly induced upon infection, and over-expression of *CML8* resulted in increased resistance [59]. 

### 3.6. Cell Wall

**Figure 5:**
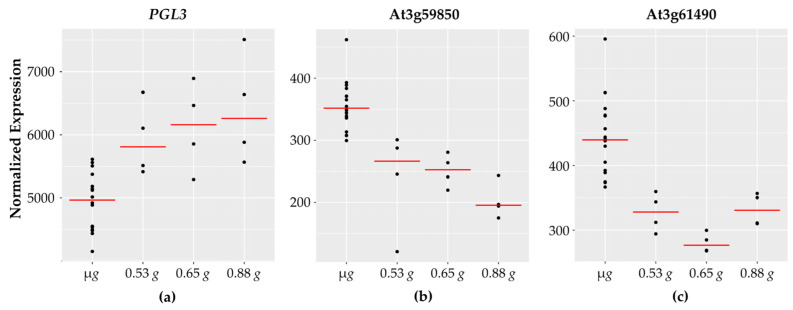
Cell wall-related genes with fractional *g* responses. Each dot on the graph shows the normalized gene expression level for the transcription factor in the sixteen microgravity replicates (µ*g*), and the four replicates at 0.53 *g*, 0.65 *g*, and 0.88 *g*. The red line indicates the mean expression level in that gravity level. Shown are three representative cell wall-associated genes: (**a**) *PGL3* which increases linearly with increasing *g* levels and two pectin lyase genes, (**b**) At3g59580 and (**c**) At3g61490, which show highest expression in microgravity and then decrease.

At least sixteen of the genes encode for enzymes involved in cell wall synthesis or modifications. Nine of the sixteen cell wall associated genes exhibit a decrease in expression with increasing *g* levels (Figure 5).

Two genes involved in cell wall synthesis cellulose synthase (namely, *CSLA2* and *CSLA3*) are opposite in their expression patterns. Beta galactosidase (*BGAL3*), glycosyl hydrolase (*GH9B8*), and polygalacturonase (*PGL3)* expression levels increase with increasing *g* levels and are lowest in microgravity. Cell wall related genes are a major category that have been reported to have altered expression in spaceflight and microgravity. These findings are consistent with the fact that reinforced plant cell walls are necessary on Earth to counteract the gravity vector and provide structural support to aerial tissues. A reduced gravity environment therefore might be expected to impact cell wall rigidity and fortification. 

Interestingly, nine of the sixteen cell wall associated genes exhibit a decrease in expression with increasing *g* levels, including two genes encoding for pectin acetylesterase (*PAE7* and *PAE12*), as well as two genes encoding for pectin lyases. Pectin is a major structural component of primary cell walls in plants with many different enzymes involved in pectin modification and breakdown. Pectin lyases [60] are enzymes that degrade pectin polymers and higher expression of pectin lyases under microgravity is suggestive of increased pectin breakdown. Pectin acetyl transferases (PAEs) reduce the degree of pectin acetylation. While *PAE7* is implicated in *Arabidopsis* growth and development and is highly expressed across different plant tissues, *PAE12* is most abundant in seeds [61]. The literature also suggests that a reduced degree of pectin acetylation is correlated with resistance to fungal pathogens [62]. 

### 3.7. Chaperone DNA J and Heat Shock Proteins

**Figure 6:**
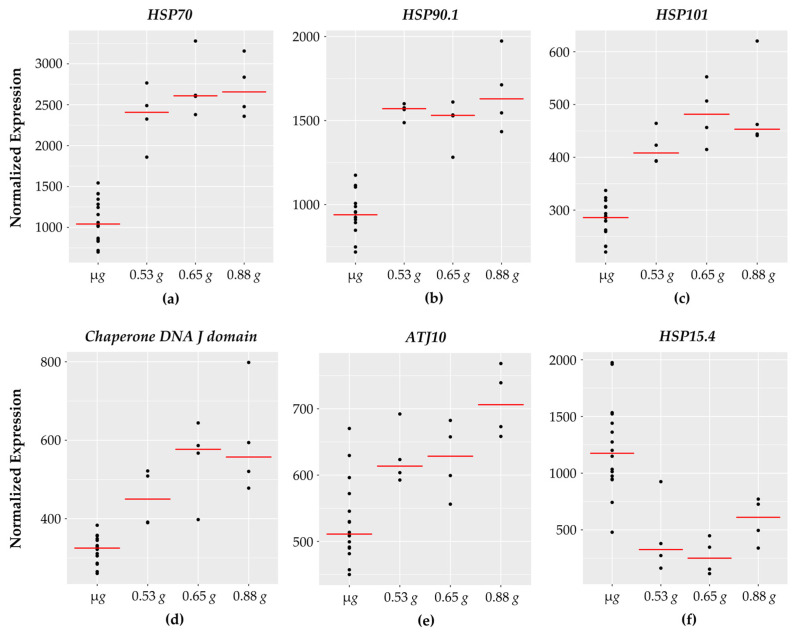
HSP genes with fractional *g* responses. Each dot on the graph shows the normalized gene expression level for the transcription factor in the sixteen microgravity replicates (µ*g*), and the four replicates at 0.53 *g*, 0.65 *g*, and 0.88 *g*. The red line indicates the mean expression level in that gravity level. Shown are five HSPs with a gravity-level responsive increase in transcription: (**a**) *HSP70*, (**b**) *HSP90.1*, (**c**) *HSP101*, (**d***) Chaperone DNA J domain*, and (**e**) *ATJ10*. In contrast, (**f**) *HSP15.4* decreases with increasing *g* with lowest expression at 0.65 *g*.

Interestingly, 9 of the 101 genes encode for heat shock and chaperone type proteins (Figure 6) with 8 of them showing increased expression with increasing *g*. It is worth noting that a heat shock responsive transcription factor, *HsfA3,* also showed increased expression with increasing *g*. One of the heat shock genes (*HSP15.4*) which belongs to the small heat shock family [63] showed downward response with higher expression at micro *g*.

As the name implies, heat shock proteins are characterized as protein chaperones with a protective function to prevent protein denaturation under high temperatures [64]. In addition to heat stress, heat shock proteins are associated with a wide range of plant stress responses including: salinity, drought, cold temperatures, flooding, heavy metal exposure, pathogen interactions, and spaceflight stressors [7,65].

### 3.8. Auxin Related

Interestingly, three genes involved in auxin transport and metabolism showed increasing expression with increasing *g*. These genes encode for an ER localized auxin transport facilitator PIN like 5 (PILS5) [66], a protein involved in regulating root auxin transport NDL2 [67], and an IAA oxidase localized in root cap cells, DAO2 [68].

### 3.9. Relationship between Gravity and Other Mechanical Stimuli

The response of plants to gravitational stimuli, touch, and mechanical stimulation share overlapping signaling pathways [69]. We therefore investigated whether the expression of the 101 fractional *g* responsive genes also respond to touch or mechanical stimulation. Surprisingly, limited overlap was detected between this dataset and touch responsive genes reported by Lee et al. [41]. The eight common genes are listed in Table S2 and include two DNA J domain chaperone proteins, a WD repeat containing transducing protein, the OSCA Ca^2+^ channel, and U-box 17. In a recent publication, Xu et al. [42] carried out a comprehensive analysis of touch responsive gene expression with a focus on identifying mitochondrial involvement. The authors subjected *Arabidopsis* seedlings to repeated mechanical stimulation (at three different time points, 0, 12, and 24 h) and compared the transcriptional response of several mutants (affected in mitochondrial function or regulation) to wild type seedlings. Comparison between the 101 genes and genes that responded to touch (in wild type and the mutants defective in mitochondrial activity) revealed an overlap of eight genes (Table S2) with 5 genes showing elevated expression at all three time points. Xu et al. also reported that several transcription factors were touch responsive either increasing or decreasing with the touch stimulus in either wild type only or a subset of the mutants. This includes eight of the twenty transcription factors in the 101 gene list (Table 2). Interestingly, there were three common genes between both touch responsive datasets [41,42] and the 101 genes reported here. Additionally, Xu et al. concluded that defense response was over-represented in their touch responsive dataset, which is consistent with our finding of several defense-related genes upregulated with increasing *g*.

Plants may also be sensitive to sound vibrations (SV)s [70]. Ghosh et al. [71] investigated differential gene expression in response to different frequencies ranging from 250–3000 Hz. The highest number of genes were responsive to 500 Hz and upregulated categories included touch responsive genes, transcription factors, and genes involved in ROS homeostasis and defense. A follow-up study [72] suggests that although SV and touch share some common signaling components, these signals may be perceived as distinct from each other. Comparison of the fractional *g* and SV responsive datasets only identified four common genes, a serine decarboxylase (*SDC1*) and *PILS5* that were upregulated by SV, and a gene involved in the response to UV-B (*RUP1*) and an ATPase (*AFG1*) that were downregulated by SV. Additionally, the fractional *g* response was opposite for each of the two genes up- and downregulated by SV. More work will be needed to explore possible relationships between partial *g* and SV.

### 3.10. Response to Hypergravity

We were particularly interested in following up on the heat shock proteins given that nine genes showed a dose-dependent upregulation with increasing *g*. HSPs have been implicated in response of plants to microgravity in both flight- and clinostat-based transcriptional studies [5,7]. Experiments with *Arabidopsis* cell cultures demonstrated upregulation of *HSP17.6A* and *HSP101* in spaceflight and prolonged clinorotation [7].

**Figure 7:**
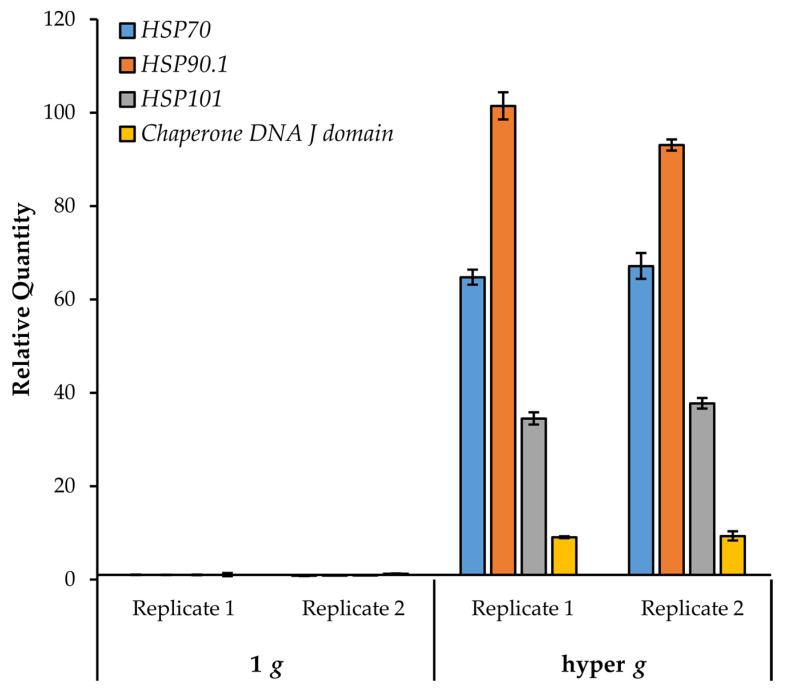
Transcriptional response of HSPs to hypergravity stimulation. HSP gene expression was monitored by quantitative RT-PCR in two replicate experiments. Mean transcriptional abundances are shown relative to the 1 *g* control. Error bars represent standard deviation of technical duplicates.

We focused on the heat shock group of genes and investigated whether their expression could be induced further by hypergravity. Seedlings (growing on agar plates) were exposed to 4 h centrifugation at 6.5× *g* in the dark. Control plates remained stationary in the dark for the same duration. Roots from control and treated seedlings were assayed for heat shock gene expression by quantitative RT-PCR. As seen in Figure 7, all of the queried heat shock genes (*HSP70, HSP90.1, HSP101*, and the *DNA J domain*) showed a robust increase in expression (> 9-fold relative to the 1 *g* stationary control). 

Interestingly, *HSP70* and *HSP90.1* were also shown to increase in response to hypergravity in *Arabidopsis* floral buds [73]. Notably, *HSP70*, *HSP90.1*, and *HSP101* were not induced by touch [41,42], although other heat shock related genes were found to be touch-responsive. These results indicate that the response of these HSPs to increasing *g* is not merely due to mechanical stimulation. Furthermore, of the 101 genes in this study, only the HSPs were found to be common with genes altered in parabolic flight [43]. Interestingly, most of the HSPs listed here were down regulated after the first sixteen parabolas [43]. Conversely, the small heat shock-related gene *HSP15.4*, which decreases with increasing *g*, was upregulated in parabolic flight (Table 2). Taken together, these data suggest that HSP genes are highly responsive to gravitational perturbations.

### 3.11. Comparison with Other Partial g Studies

Other investigators have documented transcriptional changes with partial *g* levels [6,15,16]. Similar to our study, these spaceflight experiments were also conducted in the EMCS facility and allowed for simultaneous *g* gradients with different ranges. However, the range of *g* levels between our plant signaling experiment and the other flight experiments do not overlap. Additionally, the differences in experimental conditions including lighting regimes between these experiments lead to confounding variables; therefore, direct comparisons of partial *g* responses are complicated.

Unsurprisingly, few common genes are shared between these partial-g experiments and our data set. We found 2 common transcription factor genes (*NAC97* and *GRF3*) between the 101 genes reported here and genes reported by Harranz et al. as up or down regulated under micro g compared to 1 *g* [15]. Additionally, *CNGC19* and *AT2G20142* (which encodes for a TIR domain protein), were shared between the 101 genes and genes downregulated at low-*g* (0.09 *g*) compared to 1 *g* [15]. We found no overlap in datasets with the study by Villacampa et al. which compared 1 *g* (ground) with spaceflight-simulated mars-*g* and µ*g* [16].

## 4. Conclusions

In this work, we have shown that select genes appear to respond to a narrow gradient of fractional *g* ranging from 0.53 *g* to 0.88 *g*. Six different patterns of expression were identified and GO annotation revealed that transcription, cell wall modification, biotic interactions, and heat shock responses were enriched biological functions. Many of these categories have been previously described as being responsive to the space environment. Our results also highlight that plant defense responses may be compromised in space, an unwelcome hazard that might be exacerbated if plant pathogens develop increased virulence in space as has been shown [74]. With near and long-term plans for Moon and Mars colonization, understanding plant transcriptional response to altered gravity will be particularly important. Such studies will provide valuable insight on how plants sense and respond to gravity on earth.

## Figures and Tables

**Table 1:** Gene ontology (GO) analysis of fractional *g* responsive DEGs.

GO ID	GO Term	Hit No.	Percentage	*p*-Value	FDR
GO:0006355	regulation of transcription, DNA-templated	20	17.7	7.8 × 10^−5^	0.0082
GO:0009408	response to heat	9	8.0	1.8 × 10^−5^	0.0082
GO:0009607	response to biotic stimulus	14	12.4	0.00046	0.017
GO:0022622	root system development	8	7.1	0.00074	0.021
GO:0071554	cell wall organization or biogenesis	17	15.0	0.002	0.044

**Table 2:** Enriched categories of genes based on GO analysis.

Transcription Factors							
Expression Pattern	Accession Number	Gene Symbol	Defense Related	Root Development	^a,b^ Touch Responsive	^c^ Hyper *g* Responsive	^d^ Parabolic Flight
linear up	AT1G46480	*WOX4*		+			
	AT1G69170	*SPL6*	+				
	AT2G02820	*MYB88*		+			
	AT5G23280	*TCP7*			↓		
	AT5G50820	*NAC97*			↑		
	AT5G65320	*BHLH99*			↓		
	AT5G39660	*CDF2*					
peak up	AT5G64170	*LNK1*					
threshold up	AT1G71692	*AGL12*		+			
	AT3G55370	*OBP3*	+	+			
	AT4G29930	*BHLH27*					
	AT5G03720	*HSFA3*					
	AT5G12840	*NYFA1*					
	AT5G65230	*MYB53*		+			
linear down	AT3G60630	*HM2*		+			
	AT5G15210	*ZFHD3*			↓		
	AT5G21120	*EIL2*					
threshold down	AT1G26870	*ANAC009*		+			
	AT2G36400	*GRF3*	+	+	↓		
	AT4G32890	*GATA9*			↓		
**Heat Shock Proteins**							
**Expression** **Pattern**	**Accession Number**	**Gene Symbol**	**Defense** **Related**	**Root** **Development**	^a,b^ Touch Responsive	^c^ Hyper *g* Responsive	^d^ Parabolic Flight
linear up	AT1G76700	*DNAJ (ATJ10)*			↑		down
	AT4G29920	*SMXL4*					
peak up	AT1G74310	*HSP101*					down
threshold up	AT2G20560	*DNAJ*					
	AT3G12580	*HSP70*				↑	down
	AT3G14200	*Chaperone DNA J domain*			↑		down
	AT5G03720	*HSFA3*			↑		
	AT5G52640	*HSP90.1*				↑	down
peak down	AT4G21870	*HSP15.4*					up
**Defense Related**							
**Expression** **Pattern**	**Accession Number**	**Gene Symbol**	**Defense** **Related**	**Root** **Development**	^a,b^ Touch Responsive	^c^ Hyper *g* Responsive	^d^ Parabolic Flight
linear up	AT1G11960	*OSCA1.3*	+		↑		
	AT3G26450	*Polyketide cyclase*	+				
	AT4G23570	*SGT1A*	+				
peak up	AT1G18870	*ICS2*	+				
	AT3G17690	*CNGC19*	+				
	AT4G23190	*CRK11*	+				
threshold up	AT1G29340	*PUB17*	+		↑		
	AT3G22600	*LTPG5*	+				
	AT4G37410	*CYP P450 81F4*	+				
	AT5G41180	*LRR kinase*	+				
peak down	AT4G14640	*CML8*	+				
threshold down	AT5G66590	*CAP Antigen 5*	+		↑		
**Cell Wall Related**							
**Expression** **Pattern**	**Accession** **Number**	**Gene Symbol**	**Defense** **Related**	**Root** **Development**	^a,b^ Touch Responsive	^c^ Hyper *g* Responsive	^d^ Parabolic Flight
linear up	AT3G21190	*MSR1*					
	AT5G10230	*ANN7*					
peak up	AT2G32990	*GH9B8*				↑	
	AT4G36360	*BGAL3*				↑	
threshold up	AT1G70370	*PGL3*				↑	
	AT5G22740	*CSLA2*					
	AT5G49340	*TBL4*					
linear down	AT2G34070	*TBL3*					
	AT3G59850	*Pectin lyase*					
peak down	AT1G23480	*CSLA3*					
	AT3G61490	*Pectin lyase 3*					
	AT4G19410	*PAE7*					
	AT5G67390	*Glycosyl transferase like*					
	AT1G65985	*Transmembrane protein*					
threshold down	AT3G05910	*PAE12*					
	AT3G53520	*UXS1*					

For each of the categories, accession numbers and common names of genes are listed along with their representative patterns of expression based on the grouping in Figure 1. Additional information is included on function and response as described in the text. Genes that have been associated with a particular function are indicated by a + symbol. Up or down arrows indicate the direction of the response to the stated stimulus relative to non-treated control. ^a,b^ [41] ^c^ [42] ^d^ [43].

## Data Availability

Next generation sequencing data reported in this study will be deposited to the NASA GeneLab data repository available online at https://www.genelab.nasa.gov (17 September 2021).

## Supplementary Materials

The following are available online at https://www.mdpi.com/article/10.3390/life11101010/s1, Table S1: List of fractional g responsive DEGs (Padj < 0.001) and normalized counts for all replicate samples; Table S2: Transcription Factor binding sites; Table S3: Transcription factor targets; Table S4: Overlap between fractional g responsive DEGs and touch and hypergravity datasets; Table S5: Sequence of primers used in this study.
